# The long but necessary road to responsible use of large language models in healthcare research

**DOI:** 10.1038/s41746-024-01180-y

**Published:** 2024-07-04

**Authors:** Jethro C. C. Kwong, Serena C. Y. Wang, Grace C. Nickel, Giovanni E. Cacciamani, Joseph C. Kvedar

**Affiliations:** 1 Division of Urology, Department of Surgery, University of Toronto, Toronto, ON Canada; 2 Temerty Centre for AI Research and Education in Medicine, University of Toronto, Toronto, ON Canada; 3 Harvard Medical School, Boston, MA USA; 4 USC Institute of Urology and Catherine and Joseph Aresty Department of Urology, Keck School of Medicine, University of Southern California, Los Angeles, CA USA; 5 AI Center, USC Institute of Urology, University of Southern California, Los Angeles, CA USA

**Keywords:** Medical research, Research data

## Abstract

Large language models (LLMs) have shown promise in reducing time, costs, and errors associated with manual data extraction. A recent study demonstrated that LLMs outperformed natural language processing approaches in abstracting pathology report information. However, challenges include the risks of weakening critical thinking, propagating biases, and hallucinations, which may undermine the scientific method and disseminate inaccurate information. Incorporating suitable guidelines (e.g., CANGARU), should be encouraged to ensure responsible LLM use.

In recent years, large language models (LLMs), such as generative pre-trained transformer (GPT), Large Language Model Meta AI (LLaMA), Claude, or Gemini, have demonstrated the potential to revolutionize clinical research. Indeed, it seems these disruptive technologies have now permeated nearly every aspect of the research life cycle, from generating research ideas and debugging code, to proofreading and summarizing manuscripts^1^. One of the most promising applications of LLMs is streamlining the data extraction process from unstructured texts within electronic health records. Given the demand for human-annotated data in clinical research, harnessing LLMs for this task could substantially mitigate the costs, labor, and human errors associated with manual data abstraction, thereby optimizing resource allocation and enhancing research productivity.

Extraction of pathology data presents an ideal scenario for leveraging LLMs, given the widespread availability and standardized nature of synoptic pathology reports^2^. Huang et al. recently investigated the use of ChatGPT (GPT-3.5-Turbo-16K model, version 0613) in extracting data related to pathological tumor (pT), nodal (pN), overall stage, and histology from over 900 lung cancer and pediatric osteosarcoma pathology reports^3^. The authors demonstrated that ChatGPT achieved an overall accuracy of 89%, surpassing traditional natural language processing methods such as a WordPiece tokenizer and Named Entity Recognition Classifier. Reproducibility of these results was quite robust, with an equivalence rate of 91% when these tasks were replicated a month later. Importantly, ChatGPT was fast and totaled less than $10 to use, which is substantially more cost-effective and less labor-intensive compared to hiring and training human annotators.

## Potential perils of using LLMs in clinical research

LLMs offer numerous advantages to facilitate clinical research, but they are not immune to errors. Hallucinations, characterized by fabricated responses contradicting available evidence, can pose safety and reliability concerns in clinical research settings^4^. These issues manifested in several ways within Huang et al.’s study^3^. For tumor staging, ChatGPT failed to link tumor dimensions with the correct American Joint Committee on Cancer (AJCC) 7^th^ edition pT classification in 12.6% of cases. Similarly, it incorrectly considered the total number instead of anatomical location of positive lymph nodes for nodal staging in 7.4% of cases. In instances where pathology reports were missing, ChatGPT inappropriately generated hallucinated responses in 67% of cases. Furthermore, overall staging, which is dependent on pT and pN staging, was misclassified in almost a quarter of cases due to error propagation or additional hallucinations. These findings suggest that when LLMs are applied sequentially without appropriate safeguards, hallucinations can rapidly compound and contaminate datasets.

LLM-generated responses may be colored by biases of those who create and apply them, as well as by limitations in training data, algorithm design, or policy decisions^5^. Prior studies have revealed instances where these models exhibit gender and religious biases based on the text prompts provided, further raising concerns with clinical data extraction^6,7^. Furthermore, uploading sensitive patient information to public LLMs can threaten patient privacy, necessitating appropriate regulations to ensure LLMs are Health Insurance Portability and Accountability Act (HIPAA)-compliant.

Medical research, perhaps more than any other field, requires stringent criteria for the use of new technologies, especially when they are involved in high-stakes decisions or interventions. Their outputs can greatly impact patients and their families. Therefore, they must be accurate, trustworthy, and reproducible. These requirements underscore the need for strict guidelines to ensure that all relevant information is accurately reported. Additionally, the methodology must comply with the latest guidelines to guarantee the ethical application of these technologies.

Looking beyond data abstraction, inappropriate use of LLMs may threaten the integrity of the scientific method. In light of their accessibility and speed, several groups have cautioned against the potential erosion of critical thinking skills and original thought if researchers rely on LLMs (amongst other AI-related tools) to replace rather than assist in clinical reasoning or manuscript writing^8,9^. Indeed, examples of misuse have already surfaced in the literature, where clear evidence of complete ChatGPT-generated responses (e.g., “as an AI language model,” “as of my last knowledge update,” “I don’t have access to real-time data,” “certainly, here is a possible introduction for your topic”) have been identified in published, peer-reviewed papers. Most journals have implemented their own policies governing the use of ChatGPT, generative artificial intelligence, and other large language models during the research process^10^. While these initiatives represent a positive step forward, their lack of uniformity may lead to confusion within the scientific community.

## Toward responsible and ethical use of LLMs

These concerns emphasize the need for concerted efforts to ensure responsible and ethical use of LLMs as they continue to evolve (Table 1). While pathology information has the benefit of synoptic reporting, other forms of clinical documentation are often less structured, thus may be more susceptible to hallucinations. Implementing solutions upstream of the LLM task, such as through standardized documentation templates and language, could mitigate the risk of missing data and streamline the extraction process. Others have proposed strategies in prompt design, such as a “chain-of-verification”, which entails asking the LLM a series of verification questions to allow it to correct any inconsistencies in its original response^11^. A similar approach was adopted by Huang et al., in which ChatGPT was asked to provide a degree of certainty and supporting evidence from the pathology report for each extracted attribute^3^. These techniques may help researchers identify potential vulnerabilities to hallucinations and refine their prompts to improve accuracy. In addition, LLMs should be instructed to indicate uncertainty (“I don’t know”) when no evidence is available to make appropriate inferences. The authors employed several of these strategies and should be commended for detailing their iterative prompt engineering process, underscoring the importance of transparency in both prompt design and final prompt selection.

**Table 1:** Strategies to mitigate the impact of hallucinations in large language models (LLMs)

Strategy	Description
Pre-defined purpose	Where possible, LLMs should be tailored for specific use cases (e.g. information abstraction from pathology reports) to ensure that end-users clearly understand their intended applications and limitations.
High-quality data	LLMs should be trained on domain-specific, representative, and factually accurate data. This data should not be limited to publicly available sources; efforts should also be made to include content behind paywalls or member-only subscriptions.
Data templates	Data templates facilitate data consistency and clarity, which may mitigate the risk of generating incorrect outputs.
Chain-of-verification	Chain-of-verification incorporates a structured approach for LLMs to verify each output against a reliable data source (e.g. the original pathology report) before finalization. This process enables LLMs to detect and correct any inconsistencies in their initial outputs.
Degree of uncertainty	Indicating the LLM’s confidence in its output allows end-users to better assess the reliability of the information provided and determine whether additional verification is required.
Response restrictions	Establishing “safe” boundaries for possible LLM outputs may mitigate the risk of generating incorrect or biased responses.
Human in the loop	Human oversight provides valuable domain-specific and social construct expertise to assess LLM outputs, and serves as the final safeguard against hallucinations prior to their intended use.
Updates	Processes should be established to continuously evaluate the accuracy and appropriateness of LLM responses. Updates should be provided as needed to ensure outputs remain aligned with current knowledge.

Adapted from refs. ^4,11^^,14,15^.

Despite advances in safeguarding against hallucinations, these approaches remain imperfect. GPT models, like ChatGPT, do not “reason” in the human sense, as they lack consciousness. Instead, they generate responses by analyzing patterns and relationships in the data from their training. When users pose a question, the model uses complex algorithms to predict the most likely sequence of words as a response, aiming for statistical coherence and contextual relevance. However, these models do not reason using elaborate knowledge and their capabilities are further limited by their lack of access to new data or publications after their training period. Moreover, they cannot access content behind paywalls or member-only restrictions. These limitations mean that model outputs often require a “human in the loop” to ensure accuracy and relevance. Addressing these challenges may raise additional regulatory considerations regarding the adequacy of verification measures, thresholds for acceptable error or hallucination rates, and frequency of model updates required to align with new discoveries and changes in clinical practice.

The ChatGPT, Generative Artificial Intelligence, and Natural Large Language Models for Accountable Reporting and Use (CANGARU) Guidelines is an international, multi-disciplinary effort currently working to standardize a set of recommendations for the responsible use, disclosure, and reporting of these tools in academic research and scientific discourse^12,13^. Moving forward, these guidelines may be a valuable resource for researchers, reviewers, and editorial boards, ensuring appropriate use of LLMs in clinical research.

In conclusion, LLMs like ChatGPT have reshaped the research landscape, introducing innovative and efficient approaches to data extraction, analyses, and manuscript writing. However, concerns related to hallucinations and potential biases within LLM-generated responses may jeopardize the reliability and safety of these tools. Furthermore, misuse of LLMs may detract from the integrity of the scientific method, resulting in the potential loss of critical thinking skills, particularly among junior researchers. The CANGARU guidelines are an example of how important steps are being made toward establishing an international consensus in the appropriate use of LLMs across research disciplines. By implementing guidelines and recognizing the potential harms of using LLMs in research, relevant stakeholders can uphold the principles of transparency and accountability while harnessing the full potential of LLMs in a responsible and ethical manner.
